# Perinatal Exposure of Bisphenol A Differently Affects Dendritic Spines of Male and Female Grown-Up Adult Hippocampal Neurons

**DOI:** 10.3389/fnins.2021.712261

**Published:** 2021-09-20

**Authors:** Suguru Kawato, Mari Ogiue-Ikeda, Mika Soma, Hinako Yoshino, Toshihiro Kominami, Minoru Saito, Shuji Aou, Yasushi Hojo

**Affiliations:** ^1^Department of Biophysics and Life Sciences, Graduate School of Arts and Sciences, The University of Tokyo, Tokyo, Japan; ^2^Core Research for Evolutional Science and Technology Project of Japan Science and Technology Agency, The University of Tokyo, Tokyo, Japan; ^3^Bioinformatics Project, Japan Science and Technology Agency, The University of Tokyo, Tokyo, Japan; ^4^Department of Urology, Graduate School of Medicine, Juntendo University, Tokyo, Japan; ^5^Department of Biosciences, College of Humanities and Sciences, Nihon University, Tokyo, Japan; ^6^Department of Biological Functions and Engineering, Graduate School of Life Sciences and Systems Engineering, Kyushu Institute of Technology, Wakamatsu, Japan; ^7^Department of Biochemistry, Faculty of Medicine, Saitama Medical University, Saitama, Japan

**Keywords:** Bisphenol A, perinatal exposure, spine, synapse, hippocampus, image analysis, estrous cycle

## Abstract

Perinatal exposure to Bisphenol A (BPA) at a very low dose may modulate the development of synapses of the hippocampus during growth to adulthood. Here, we demonstrate that perinatal exposure to 30 μg BPA/kg per mother’s body weight/day significantly altered the dendritic spines of the grownup rat hippocampus. The density of the spine was analyzed by imaging of Lucifer Yellow-injected CA1 glutamatergic neurons in adult hippocampal slices. In offspring 3-month male hippocampus, the total spine density was significantly decreased by BPA exposure from 2.26 spines/μm (control, no BPA exposure) to 1.96 spines/μm (BPA exposure). BPA exposure considerably changed the normal 4-day estrous cycle of offspring 3-month females, resulting in a 4∼5 day estrous cycle with 2-day estrus stages in most of the subjects. In the offspring 3-month female hippocampus, the total spine density was significantly increased by BPA exposure at estrus stage from 2.04 spines/μm (control) to 2.25 spines/μm (BPA exposure). On the other hand, the total spine density at the proestrus stage was moderately decreased from 2.33 spines/μm (control) to 2.19 spines/μm (BPA exposure). Thus, after the perinatal exposure to BPA, the total spine density in males became lower than that in females. Concerning the BPA effect on the morphology of spines, the large-head spine was significantly changed with its significant decrease in males and moderate change in females.

## Introduction

Environmental low-dose exposure to endocrine disrupters has emerged as a risk factor for brain function. Bisphenol A (BPA, synthetic material of polycarbonate resin used in dental prostheses, sealants, compact discs, and baby bottles) is a typical xenoestrogen.

The toxic effects of high-dose (mg/kg body weight/day) BPA have been investigated about development and functions of the reproduction systems (Fisher et al., 1999; Al-Hiyasat et al., 2002; Grote et al., 2004; Halldin et al., 2005). However, low-dose exposure (μg/kg/day) to BPA shows rather weak toxic effects on reproductive or endocrine functions in the peripheral tissues, probably due to the efficient detoxification of chemicals by the liver. On the other hand, low-dose exposure to BPA may seriously affect brain function (Quesada et al., 2002; Negishi et al., 2003; Kawato, 2004; Mukai et al., 2006; Ogiue-Ikeda et al., 2008; Hasegawa et al., 2013). The low-dose effects of BPA on dendritic spines have often been investigated in adult rats. For example, BPA injection (s.c. injection) to estradiol (E2)-supplemented ovariectomized (OVX) female adult rats inhibits the E2-induced increase of the spine-synapse density in the hippocampus and prefrontal cortex (Leranth et al., 2008). BPA exposure over a 4-week period in adult monkey also inhibits the E2-induced increase of the spine synapse-density in the hippocampus (Leranth et al., 2008). In these experiments, BPA probably penetrates into the brain by crossing the blood-brain barrier even in adult animals.

Upon perinatal exposure to BPA, BPA can penetrate much more easily into the brain of embryo and fetus where the blood-brain barrier is not completely developed (Uchida et al., 2002). The detoxification of BPA in the brain is probably very weak due to the extremely low level of drug-metabolizing enzymes in the brain (Miksys and Tyndale, 2002; Kishimoto et al., 2004; Chinta et al., 2005). Therefore, when animals are perinatally exposed to BPA, a low level of BPA might be adequate to exert its low-dose effect on the development of the brain’s neural circuit.

In a behavioral study of perinatal BPA exposure, fetal or neonatal exposure to BPA inhibits sexual differentiation of non-reproductive behaviors, including performances in the Morris water maze test, open field test, elevated plus maze test, passive avoidance test, and forced swimming test (Carr et al., 2003; Kubo et al., 2003; Fujimoto et al., 2006) at doses as low as 1/1000 of those required for the stimulation of uterine growth (Ashby, 2001).

Because the density and morphology of spines of the hippocampal CA1 neuron are positively correlated with spatial learning and memory, many investigations were performed in adult animals to demonstrate that BPA alters hippocampal synapses (MacLusky et al., 2005; Leranth et al., 2008; Hajszan and Leranth, 2010; Inagaki et al., 2012; Tanabe et al., 2012). To solve the paradoxical problem of too low affinity of BPA with estrogen receptor alpha (ERα) and estrogen receptor beta (ERβ), specific BPA receptor estrogen receptor–related protein gamma (ERRγ) was identified as a high-affinity BPA receptor in spine modulation by BPA in hippocampal neurons (Tanabe et al., 2012).

Recently, several studies have focused on the effects of perinatal BPA exposure on the neural circuits, such as hippocampal dendritic spines of monkeys (Elsworth et al., 2013) and rats (Liu et al., 2015, 2016) because BPA-induced alteration in brain development may cause neurodegeneration, resulting in not only memory and cognitive disorders but also Parkinson’s disease (Masuo and Ishido, 2011) and autism spectrum (de Cock et al., 2012).

There are still insufficient investigations about the effects of perinatal BPA exposure on the spine density and morphology of hippocampal neurons of grownup offspring. In the current study, BPA was administrated to fetal and newborn rats via their mother’s oral route. Mother rats were administered BPA approximately 30 μg/kg body weight/day, beginning at the 15th day of pregnancy (gestational day 15, GD15) until their offspring had reached post neonatal day 7 (PD7). We investigate endocrine disrupting actions of low doses of BPA during the development of the hippocampus by measuring dendritic spine density and morphology of grownup offspring at age 3 months.

## Materials and Methods

### Chemicals

Bisphenol A and Lucifer Yellow were purchased from Sigma (United States). Other chemicals were purchased from FUJIFILM-Wako (Japan).

### Animals

Pregnant Wistar (Charles River) female rats at gestational day 10 (GD10) were purchased from Kyudo Corp. (Japan) and were housed in the animal house of the University of Tokyo. Mother rats were exposed to BPA, and their offspring were raised until they grew to 10 weeks old. The animals were housed in polycarbonate cages with free access to food and water in an air-conditioned room under a 12:12-h light and dark cycle (light period 08:00-20:00 h). The experimental procedure of this research was approved by the Committee for Animal Research of the University of Tokyo.

### Perinatal BPA Exposure

Bisphenol A was dissolved in dimethyl sulfoxide (DMSO) as stock solution and then dissolved in distilled water to a final concentration of 0.1 ppm and orally administered to mother rats as drinking water from GD15 to postneonatal day 7 (PD7) for 2 weeks. Control rats were treated under the same condition except for BPA. Mother rats were administered BPA approximately 30 μg/kg body weight/day for this period and after PD7, and they were allowed tap water *ad libitum*. Mother rats treated with BPA gave birth to six female and six male offspring on average. On PD21, all offspring were weaned and then classified and housed in the same sex and litter groups and were allowed food and tap water *ad libitum* until the end of the experiment. All offspring were raised until they grew to 10 weeks old.

### Estrous Cycle

The estrous cycle of grownup offspring female rats aged 8 weeks was monitored for 2 weeks prior to the experiments to ensure that all subjects were cycling regularly. The stage of the estrous cycle was determined using a lavage technique. Characterization of vaginal cells was based on the description by Turner and Bagnara. All grownup control female rats showed the regular 4-day estrous cycle: proestrus, estrus, diestrus-1, and diestrus-2. However, BPA exposure considerably changed the normal estrous cycle, resulting in a 4∼5 day estrous cycle with 2 days estrus stages in most of the subjects (Table 1).

**TABLE 1:** Typical estrus cycle of control and BPA-exposed female rats.

Control	Days-								
Female 1	E	D1	D2	P	E	D1	D2	P	E
Female 2	E	D1	D2	P	E	D1	D2	P	E
Female 3	E	D1	D2	P	E	D1	D2	P	E
Female 4	E	D1	D2	P	E	D1	D2	P	E
Female 5	E	D1	D2	P	E	D1	D2	P	E
Female 6	E	D1	D2	P	E	D1	D2	P	E

**BPA**	**Days-**								

Female 1	E	E	D1	D2	P	E	E	D1	D2
Female 2	E	E	D1	D2	P	E	E	D1	P
Female 3	E	E	D1	D2	P	E	E	D1	P
Female 4	E	E	D1	P	E	E	D1	D2	P
Female 5	E	E	D1	D2	P	E	E	D1	P
Female 6	E	D1	D2	P	E	E	D1	P	E

*Abbreviations used are: Proestrus (P), Estrus (E), Diestrus-1 (D1), and Diestrus-2 (D2).*

### Preparation of Hippocampal Slices

Hippocampal slices were prepared from 12-week-old male and female rats that were deeply anesthetized and perfused transcardially with PBS (0.1 M phosphate buffer and 0.14 M NaCl, pH 7.3), followed by a fixative solution of 3.5% paraformaldehyde. Immediately after decapitation, the brain was removed from the skull and post-fixed with the fixative solution. The dorsal hippocampus was then dissected and 400-μm-thick transverse slices to the long axis from the middle third of the hippocampus were prepared with a vibratome (Dosaka, Japan).

### Imaging and Analysis of Spines

#### Current Injection of Lucifer Yellow

Neurons within slices were visualized by an injection of Lucifer Yellow under a Nikon E600FN microscope (Japan) equipped with a C2400-79H infrared camera (Hamamatsu Photonics, Japan) and with a 40 × water lens (Nikon). Dye was injected with a glass electrode whose tip was filled with 5% Lucifer Yellow for 15 min, using Axopatch 200B (Axon Instruments, United States). Approximately five neurons within a 10-30-μm depth from the surface of a slice were injected.

#### Confocal Laser Microscopy and Analysis

The imaging was performed from sequential z-series scans with a confocal laser scan microscope (LSM5; Carl Zeiss, Germany) at high zoom (×3.0) with a 63× water immersion lens, NA 1.2. For Lucifer Yellow, the excitation and emission wavelengths were 488 and 515 nm, respectively. For analysis of spines, a three-dimensional image was reconstructed from approximately 40 sequential z-series sections every 0.45 μm. The applied zoom factor (×3.0) yielded 23 pixels per 1 μm. The z-axis resolution and the lateral resolution were approximately 0.47 and 0.16 μm, respectively. Our resolution limits were regarded to be sufficient to allow the determination of the spines. Confocal images were then deconvoluted using AutoDeblur software (AutoQuant, United States).

The density of spines as well as the head diameter were analyzed with Spiso-3D (automated software calculating geometrical parameters of spines) developed by the Bioinformatics Project of Kawato’s group (Mukai et al., 2011). Spiso-3D has nearly an equivalent capacity as Neurolucida (MicroBrightField, United States) which, however, needs time-consuming manual operation. The single apical dendrite was analyzed separately. These dendrites were present within the stratum radiatum between 100 and 200 μm from the pyramidal cell body in the stratum radiatum of the CA1 region. The spine density was calculated from the number of spines along secondary dendrites having a total length of approximately 50 μm.

Spine shapes were classified into three categories as follows: (1) a small-head spine, whose head diameter is smaller than 0.4 μm; (2) a middle-head spine, which has a 0.4- to 0.5-μm spine head; and (3) a large-head spine, whose head diameter is larger than 0.5 μm.

Small-, middle-, and large-head spines probably have different numbers of α-amino-3-hydroxy-5-methyl-4-isoxazolepropionic acid (AMPA) receptors, and thereby, these three types of spines may have different efficiency in memory storage. The number of AMPA receptors (including GluR1 subunits) in the spine increases as the size of post-synapse increases, whereas the number of N-methyl-D-aspartate (NMDA) receptors (including NR2B subunits) might be relatively constant (Shinohara et al., 2008).

These three categories were useful to distinguish different responses to BPA treatments.

Because the majority of spines (>95%) had a distinct head and neck, we analyzed spines having a distinct head.

### Statistical Analysis

For statistical analysis of the total spine density in Figure 2, we employed two-way ANOVA with two factors, including BPA (two groups: control and BPA) and sex categories (three groups: male, female proestrus, and female estrus), followed by a Tukey–Kramer multiple comparison test.

For statistical analysis of large-head spine density in Figure 6, we employed one-way ANOVA with sex categories (male, female proestrus, and female estrus), followed by a Tukey–Kramer multiple comparison test.

## Results

### Perinatal BPA Exposure Alters Hippocampal CA1 Dendritic Spines in Offspring Adult Male and Female Rats

**FIGURE 1:**
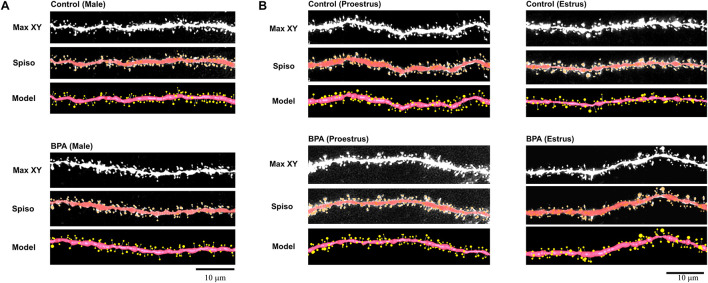
Image analysis demonstrates the changes of the morphology and density of spines upon perinatal exposure of BPA in the hippocampus of grownup male **(A)** and female rats **(B)**. **(A)** Male controls (control, male) and BPA-exposed males (BPA, male) are shown. Spine images along the secondary dendrites in the stratum radiatum. Maximal intensity projections onto the XY plane from z-series confocal micrographs (Max XY). Spine images analyzed by Spiso-3D (Spiso) and three-dimensional model illustration (Model). **(B)** Female controls at proestrus (control, proestrus), BPA-exposed females at proestrus (BPA, proestrus), female controls at estrus (control, estrus) and BPA-exposed females at estrus (BPA, estrus) are shown. Spine images along the secondary dendrites in the stratum radiatum. Maximal intensity projections onto the XY plane from z-series confocal micrographs (Max XY). Spine images analyzed by Spiso-3D (Spiso) and three-dimensional model illustration (Model). Scale bar = 10 μm.

To determine the effect of very low-dose perinatal BPA exposure (30 μg/kg/day to dam, GD15-PD7) on hippocampal CA1 spine density and morphology in grownup adult male and female rats, we analyzed secondary branches of the dendrites of the stratum radiatum. Images of dendritic spines after Spiso-3D analysis are shown in Figures 1A (male), B (female).

### Comparison of the Total Spine Density Between Males and Females

**FIGURE 2:**
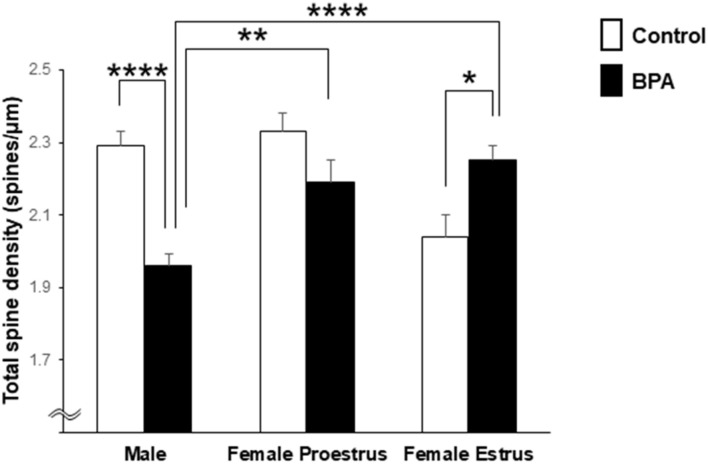
Comparison of the total density of spines in the hippocampal CA1 neurons of grownup male and female rats after perinatal BPA exposure. Results are represented as mean ± SEM. In each group, the white and black columns express the control and BPA-exposure data, respectively. We investigated 6 rats, 13 slices, 25 neurons, 50 dendrites, ∼5700 spines for male control; 8 rats, 13 slices, 26 neurons, 51 dendrites, ∼5000 spines for male BPA; 3 rats, 7 slices, 13 neurons, 26 dendrites, ∼3000 spines for female proestrus control; 4 rats, 8 slices, 16 neurons, 32 dendrites, ∼3500 spines for female proestrus BPA; 3 rats, 8 slices, 16 neurons, 31 dendrites, ∼3200 spines for female estrus control; and 6 rats, 15 slices, 30 neurons, 60 dendrites, ∼6800 spines for female estrus BPA. Statistical analysis was performed with two-way ANOVA followed by Tukey–Kramer multiple comparison test. Statistical significance yielded *p < 0.05, **p < 0.01, and ****p < 0.001. For two-way ANOVA, two factors include BPA (control, BPA) and sex categories (male, female proestrus, and female estrus).

Comparison of the total density of spines of grownup male and female rats was performed with two-way ANOVA followed by a Tukey–Kramer multiple comparison test (see Figure 2). Two-way ANOVA with two factors including BPA (two groups: control and BPA) and sex categories (three groups: male, female proestrus, and female estrus) showed an effect of BPA [*F*(1,244) = 5.018, *p* = 0.026] and sex categories [*F*(2,244) = 4.806, *p* = 0.009], and a BPA × sex category interaction [*F*(2,244) = 16.999, *p* = 1.22E-07] (Figure 2).

In male rats, perinatal BPA exposure resulted in a significant reduction in the total spine density from 2.29 spines/μm in male control rats to 1.96 spines/μm in male BPA exposed rats (*p* < 0.001, Tukey–Kramer multiple comparison test) (Figure 2).

In control female rats, the total spine density significantly oscillated across the estrous cycle, peaking at 2.33 spines/μm at proestrus and reducing to 2.04 spines/μm at estrus (Figure 2). These oscillation results were consistent with our earlier results (Kato et al., 2013).

In female rats at proestrus, BPA exposure moderately reduced the total spine density, but not significantly, with 2.33 spines/μm (control) and 2.19 spines/μm (BPA exposed) (Figure 2).

In females at estrus, however, perinatal BPA exposure significantly increased the total spine density from 2.04 spines/μm (control) to 2.25 spines/μm (BPA exposed) (*p* < 0.05, Tukey–Kramer multiple comparison test) (Figure 2).

### Comparison of the Spine Head Diameter Between Male and Female

**FIGURE 3:**
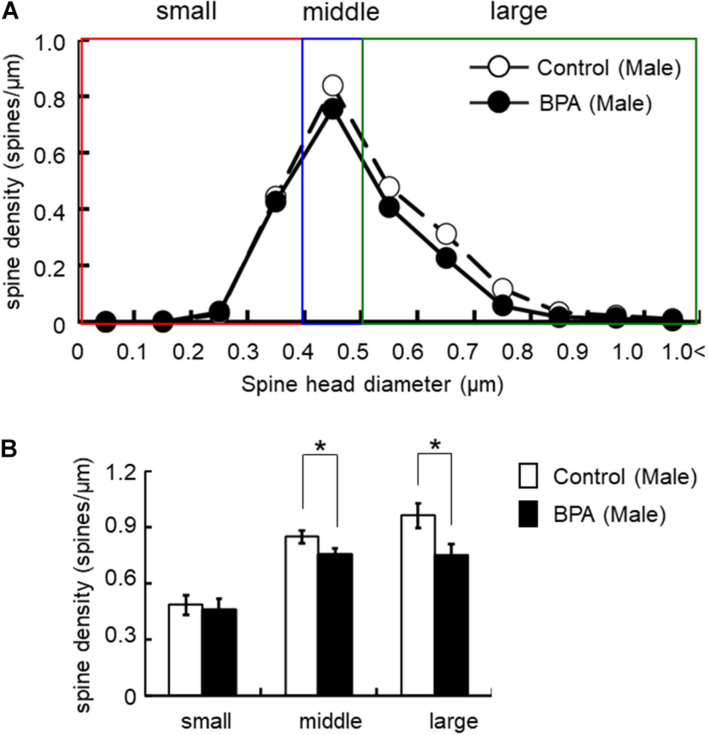
Changes in the morphology of spines upon perinatal exposure of BPA in the hippocampus of grownup male rats. **(A)** Histogram of spine-head diameters without BPA (control, open circle) or with BPA (BPA, filled circle) in male rats. **(B)** Density of three subtypes of spines, from left to right: small-, middle-, large-head spines. Results are represented as mean ± SEM. In each group, the white and black columns are expressed for the control and BPA-treated data, respectively. We investigated 6 rats, 13 slices, 25 neurons, 50 dendrites, ∼5700 spines for male control and 8 rats, 13 slices, 26 neurons, 51 dendrites, ∼5000 spines for male BPA. The significance of the BPA effects was confirmed via statistical analysis using *t-*tests. Statistical significance yielded *p < 0.05.

**FIGURE 4:**
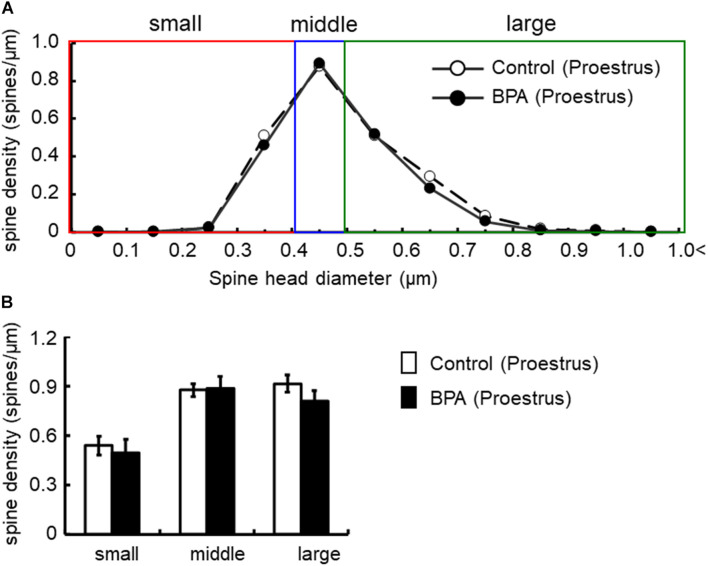
Changes in the density of spines in the hippocampus of grownup female rats at proestrus stage by perinatal BPA exposure. **(A)** Histogram of spine-head diameters without BPA (control, open circle) or with BPA (BPA, filled circle) in female rats at proestrus stage, respectively. **(B)** Density of three subtypes of spines, from left to right: small-, middle-, large-head spines. Results are represented as mean ± SEM. In each group, the white and black columns are expressed for the control and BPA-treated data, respectively. We investigated 3 rats, 7 slices, 13 neurons, 26 dendrites, ∼3000 spines for female proestrus control and 4 rats, 8 slices, 16 neurons, 32 dendrites, ∼3500 spines for female proestrus BPA. The significance of the BPA effects was confirmed via statistical analysis using *t*-tests.

**FIGURE 5:**
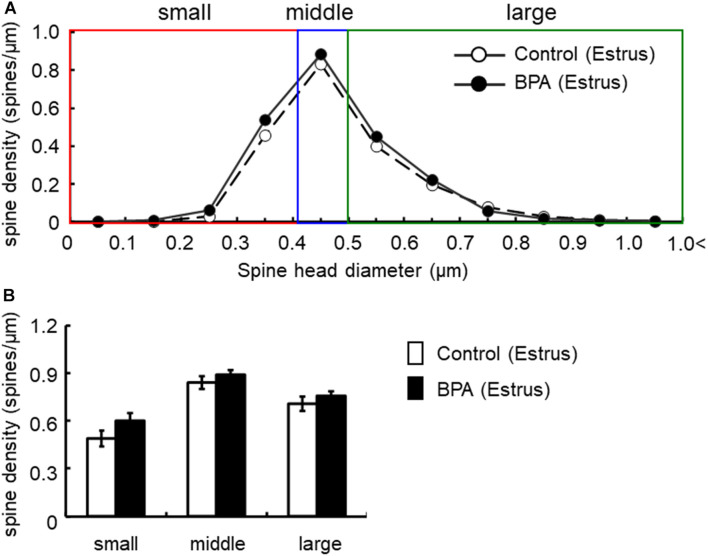
Changes in the density of spines in the hippocampus of grownup female rats at estrus stage by perinatal BPA exposure. **(A)** Histogram of spine-head diameters without BPA (control, open circle) or with BPA (BPA, filled circle) in female rats at estrus stage, respectively. **(B)** Density of three subtypes of spines, from left to right: small-, middle-, large-head spines. Results are represented as mean ± SEM. In each group, the white and black columns are expressed for the control and BPA-treated data, respectively. We investigated 3 rats, 8 slices, 16 neurons, 31 dendrites, ∼3200 spines for female estrus control and 6 rats, 15 slices, 30 neurons, 60 dendrites, ∼6800 spines for female estrus BPA. The significance of the BPA effects was confirmed via statistical analysis using *t*-tests.

For morphological analysis, a histogram of spine head diameter distribution is shown in Figure 3 (male), Figure 4 (female proestrus), and Figure 5 (female estrus).

For statistical analysis in males, spines were classified as large- (0.96 spines/μm), middle- (0.85 spines/μm), and small-head spines (0.48 spines/μm) (Figure 3). Perinatal BPA treatment significantly altered the spine-head diameter distribution by decreasing large-head spines from 0.96 spines/μm to 0.75 spines/μm (*p* < 0.05, *t-*test) and middle-head spines from 0.85 spines/μm to 0.75 spines/μm (*p* < 0.05, *t*-test) (Figure 3). The density of small-head spines was not significantly affected by perinatal BPA exposure (0.46∼0.48 spines/μm).

In females at proestrus, BPA exposure did not significantly alter the head diameter distribution and three subpopulations distribution of large-, middle-, and small-head spines (Figure 4). In control and BPA-exposed females at proestrus, spines were classified as large-head spines (in control 0.92 spines/μm, in BPA exposed 0.81 spines/μm), middle-head spines (in control 0.88 spines/μm, in BPA exposed 0.89 spines/μm), and small-head spines (in control 0.54 spines/μm, in BPA exposed 0.49 spines/μm).

In females at estrus, when we take a close look at three subpopulations’ density of large-, middle-, and small-head spines, a significant increase was not observed although a significant increase was observed as a total density of spines (Figure 5). In control and BPA-exposed females at estrus, spines were classified as large-head spines (in control 0.71 spines/μm, in BPA 0.76 spines/μm), middle-head spines (in control 0.84 spines/μm, in BPA 0.89 spines/μm), and small-head spines (in control 0.49 spines/μm, in BPA 0.60 spines/μm) (Figure 5).

**FIGURE 6:**
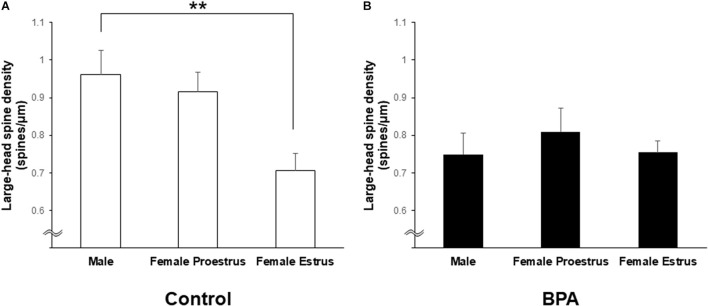
Changes in the density of large-head spines in the hippocampus of grownup male and female rats by perinatal BPA exposure. **(A)** Comparison of the density of the large-head spines between male, female at proestrus, and female at estrus without perinatal BPA exposure (Control). **(B)** Effect of BPA exposure on the large-head spine density of male, female at proestrus, and female at estrus with perinatal BPA exposure (BPA). Results are represented as mean ± SEM. Statistical analysis was performed Tukey–Kramer multiple comparison test when one-way ANOVA showed significant difference. Statistical significance yielded, **p < 0.01.

Large-head spines could have highest memory capacity among the three subtypes of spines (small-, middle-, and large-head spines) because large-head spines may have the highest number of AMPA receptors (Shinohara et al., 2008). Interestingly, after perinatal BPA exposure, the difference in the large-head spine density between males and females at estrus disappeared although a significant difference was observed in control (without BPA) between males and females at estrus (see Figure 6).

## Discussion

This study demonstrates that perinatal exposure to BPA via mother rats at a daily dose of 30 μg/kg decreased spine density in the hippocampal CA1 region in male grownup offspring. On the other hand, the BPA exposure increased spine density in estrus females and moderately decreased in proestrus females.

On the basis of the no-observed-adverse-effect level (NOAEL), the tolerable daily intake (TDI), which is the maximum acceptable dose in humans, was calculated as 50 μg/kg per day (EFSA Panel on Food Contact Materials, Enzymes, Flavourings, Aids, 2010) and 4 μg/kg per day (EFSA Panel on Food Contact Materials, Enzymes, Flavourings, Aids, 2015). The daily intake of BPA in the current experiment (30 μg/kg per day) was under the TDI in 2010. The human TDI is the safety index and any dosage under the TDI is not supposed to cause adverse effects. Because, in the current study, BPA induced changes in the spine density in hippocampus at the dosage below the TDI established in 2010 by the EFSA, our results support the latest update of this TDI (EFSA Panel on Food Contact Materials, Enzymes, Flavourings, Aids, 2015). It should be noted that apparent abnormal effects of BPA exposure in the current study are an alteration of the estrous cycle. BPA-exposed female rats showed a 4∼5 day estrous cycle with a 2-day estrus stage in most of the subjects, which is clearly different from a normal 4-day estrous cycle of healthy control female rats.

### Correlation of BPA Effects Between Spines and Behaviors

Hippocampal CA1 spine density of male and female rats were significantly different depending on stages of the female estrous cycle, consisting of proestrus, estrus, diestrus-1, and diestrus-2 (Kato et al., 2013). The total spine density of males was greater than that of females at the estrus stage but the same as that at proestrus. Perinatal BPA exposure in males decreased the total spine density and the density of large-head spine (Figures 2, 6). On the other hand, perinatal BPA exposure in females significantly increased the total spine density at the estrus stage and moderately decreased it at the proestrus stage (Figures 3, 4, 6).

These differences may be caused by the interactions between BPA and different levels of E2 in the hippocampus of male, female proestrus, and female estrus states. From our earlier study (Kato et al., 2013), the hippocampal level of E2 was ∼8.4 nM (male), ∼4.3 nM (proestrus), and ∼1 nM (estrus). The total spine density in the current study was 2.29 spines//μm (male), 2.33 spines//μm (proestrus), 2.04 spines/μm (estrus). These hippocampal spine densities are nearly proportional to the E2 level. BPA application might reduce the total spine density of the hippocampus in a high E2 condition and may increase it in a low E2 condition. These considerations of BPA-E2 interactions may be supported by several earlier studies that indicate application of BPA blocked the E2 application-induced increase of the spine-synapses in OVX female rats in which endogenous hippocampal E2 was largely depleted (MacLusky et al., 2005).

The observed moderate changes in the spine density (∼10%) could be sufficient to modify synaptic plasticity, such as the long-term potentiation (LTP). From our earlier investigations, supplementation of E2 to acute hippocampal slices (sex hormone–depleted slices), the observed maximum increase of the spine density was around 12∼20% (Ooishi et al., 2012; Hasegawa et al., 2015; Hatanaka et al., 2015). The electrophysiological investigations showed that 10 nM E2 perfusion did induce LTP even with weak theta-burst stimulation in the same acute hippocampal slices (Hasegawa et al., 2015).

Hippocampal CA1 neurons and synapses are known to be closely correlated with spatial learning and memory. Earlier studies demonstrate sex differences in spatial learning and memory by means of Water maze test (D’Hooge and De Deyn, 2001). Perinatal BPA exposure decreased sex differences of spatial learning and memory at adulthood of offspring rats because perinatal BPA exposure impaired the performance of males but not females (Carr et al., 2003; Xu et al., 2007). These results may have some correlation with the current observation of the spine density decrease in males and spine increase at female estrus. OVX induced impairment of behavior with the water maze test (Talboom et al., 2008), and this could be due to OVX-induced decrease in spine density.

Recent studies focusing on the effects of perinatal plus direct postnatal BPA exposure (150 μg BPA/kg/day) on hippocampal dendritic spines of rats demonstrated that male spine density decreased significantly and female spine density decreased moderately (Liu et al., 2015, 2016). Several points should be paid attention in these studies. The examination of female spines was performed without discrimination of estrous cycle stages, and the golgi stained spine images were two-dimensional projection images without sufficient quality (Liu et al., 2015, 2016). Theoretically two-dimensional projection images have artificially shorter dendrite length than the original 3-dimensional images, resulting in an incorrect estimation of the spine density.

An estrous cycle–dependent cyclic fluctuation of the spine density in the hippocampal CA1 neuron is known from earlier studies (Woolley et al., 1990). The density of total spines decreased by going from proestrus to estrus by roughly 30%, which is nearly identical to the current observation (Figure 2; Kato et al., 2013). In addition, the density of large-head spines was decreased from proestrus to estrus (Kato et al., 2013).

On the basis of estrous cycle–dependent cyclic oscillation of CA1 spine density, spatial ability would vary across the estrous cycle with females in proestrus outperforming those in estrus. Many earlier investigations indicate the influence of estrous cycle stages on spatial learning and memory by means of the Morris water maze test. Memory retention of the Morris water maze varies significantly across the estrous cycle of the female rat with females in the proestrus stage outperforming those in the estrus stage (Frye, 1995; Warren and Juraska, 1997). Our current analysis of spine density is able to detect the estrous cycle–dependent difference of female hippocampal spines. Spine density analysis is found to be one of the most sensitive methods to detect the estrous cycle–dependent difference in the female hippocampus.

The effects of perinatal plus direct postnatal BPA exposure (150 μg BPA/kg/day) on the hippocampus-dependent behavior, using the Morris water maze, demonstrate that BPA significantly impaired spatial memory of both male and female offspring 3-month-old rats (Liu et al., 2015, 2016). Perinatal BPA exposure was performed via drinking water (150 μg BPA/kg/day) for mother and additional direct BPA exposure (150 μg BPA/kg/day) for pups was performed through drinking water until rats became 3 months old for the Water maze test. Investigations of the female Water maze task were performed without discrimination of estrous cycle stages (Liu et al., 2016).

Hippocampus-dependent memory and learning is also modulated by subcortical cholinergic, dopaminergic, and serotonergic systems. Perinatal exposure to BPA in mice induced memory impairment as measured by a step-through avoidance test, which was associated with a dramatic reduction in the cholinergic innervation of the hippocampus at 7-week-old mice (Miyagawa et al., 2007).

### Estrogen Receptor–Related Protein Gamma Is a Candidate of BPA Receptor

Searching for BPA receptors is an important and difficult problem. Although ERα was assumed to be a receptor of BPA in earlier studies, the binding affinity of BPA to ERα is much lower (around 1/100–1/2000) than that of E2 (Kuiper et al., 1997; Morohoshi et al., 2005), which is consistent with our binding assay results. Therefore, low-dose BPA probably cannot induce significant effects on spinogenesis through ERα.

Bisphenol A binds strongly to ERRγ with IC_50_ of 13 nM though E2 does not bind to ERRγ (Takayanagi et al., 2006). In our earlier study (Tanabe et al., 2012), we found the BPA-induced rapid increase of spines in hippocampal acute slices. We indicated that ERRγ in CA1 glutamatergic neurons regulates these BPA effects of spine modulation. To obtain these conclusions, we used OH-Tamoxifen (OH-Tam), an antagonist of ERRγ, ERα (estrogen receptor alpha), and ERβ (estrogen receptor beta) (Fitts et al., 2011), and ICI, an antagonist of ERα/ERβ (Tanabe et al., 2012). OH-Tam completely suppressed the increase of spines by BPA; however, ICI did not suppress the BPA-induced increase of spines. Therefore, we could conclude that ERRγ is a high-affinity receptor for BPA in the isolated hippocampal slice system. In hippocampal slices, ERRγ was significantly expressed in pyramidal neurons of CA1 and CA3, and granule cells of dentate gyrus (DG) as judged from immunostaining and Western blot analysis (Tanabe et al., 2012). Similar investigations using OH-Tam/ICI were performed in hippocampal primary cultured neurons, and the ERRγ-mediated mechanism of BPA action was observed on glutamate-induced Ca influx (Zhong et al., 2019).

Estrogen receptor–related protein gamma might also play a significant role as a BPA receptor in the current *in vivo* BPA experiments, which include neuronal developmental processes of the whole brain. However, the *in vivo* neural circuit is too complex to analyze at the moment. One reason is that we do not have a specific antagonist of ERRγ. Furthermore, a combination of Tam/ICI inhibitors is not applicable to *in vivo* systems because ICI cannot cross the blood-brain barrier to reach the brain after s.c. injection although Tamoxifen can cross the blood-brain barrier for blocking of ERRγ/ERα/ERβ in the brain. Another reason is that neural circuits around the hippocampus may include several different ERRγ-expressing neurons that project to the hippocampus from the other brain regions in addition to hippocampal glutamatergic neurons (see Supplementary Figure 1).

The anatomical neural circuits are very different between the hippocampus *in vivo* and isolated hippocampal slices. As shown in Supplementary Figure 1, the hippocampus *in vivo* receives complicated projections of cholinergic neurons from medial septum/diagonal band of Broca (MSDB), serotonergic neurons from median Raphe (MR), and glutamatergic neurons from the supramammillary area (SUM). In addition, GABAergic projections are present. These neurons express ERα or ERβ (Leranth et al., 2000). ERRγ is expressed in MSDB (Lorke et al., 2000), but ERRγ expression in the MR region is not clear. These neurons project to GABA neurons and pyramidal neurons (PY) in the hippocampus.

Because of these complex neural circuits, the explanation of the mechanisms is very difficult for *in vivo* BPA effects, for example, BPA-induced suppression of E2-induced increase in hippocampal spines. A typical example is the following *in vivo* study. In OVX female adult rats, the s.c. injection of E2 rapidly increased the spine-synapse density in the hippocampus. Although application of only BPA did not affect the spine-synapse density, co-application of BPA with E2 suppressed the E2-induced increase in the spine-synapse density (MacLusky et al., 2005). These processes may include cholinergic or serotonergic neurons, which project to the hippocampus from the subcortical region because the removal of cholinergic or serotonergic neuron projections decreased E2 effects (Leranth et al., 2000, 2003).

Several possible signaling pathways can be considered for explanation of these *in vivo* events. (Pathway A) E2 activates cholinergic or serotonergic neurons → activating GABA neurons → suppressing PY. (Pathway B) E2 activates cholinergic neurons→ activating PY. (Pathway C) BPA suppresses E2 action within cholinergic neurons (via ERRγ) → BPA and E2 act only in PY and GABA neurons. (Pathway D) BPA does not suppress E2 action in cholinergic neurons → E2 activating GABA neurons → suppressing PY. (Pathway E) Direct BPA and E2 actions in intra-hippocampal GABA neurons. (Pathway F) Direct BPA and E2 actions within PY.

On the other hand, in the isolated hippocampal slices, we can focus on PY and GABA neurons. Because ERRγ was expressed in PY whose population was much greater than that of GABA interneurons, the effect of BPA on hippocampal slices was primarily caused by the BPA action on PY.

### Perinatal BPA Exposure May Affect the Development of the Offspring Hippocampus

In the current study, perinatal BPA exposure changed the spine density of CA1 glutamatergic neurons in the hippocampus of grownup rats. This result suggests that perinatal BPA exposure may affect the development of the offspring brain because the offspring were exposed to BPA only through placental blood circulation and breast milk from their mothers, and none of the offspring were exposed to any BPA after weaning.

Iguchi and coworkers demonstrated that BPA, injected into the mouse mother’s body (single s.c. injection), was transferred to the brains of fetus within 1 h *via* going through the immature blood-brain barrier (Uchida et al., 2002). In contrast to the efficient detoxification of endocrine disrupters, such as BPA in the liver, detoxification in the brain may be less efficient due to the low level of drug-metabolizing enzymes in the brain (Miksys and Tyndale, 2002; Miksys et al., 2002). These findings suggest that BPA penetrates into mammalian brains (including human brains) at concentrations sufficient to impact brain development and function.

Perinatal BPA exposure in mice decreased the number of neural progenitor cells and granule cells in the hippocampal DG on postnatal day 70 (Komada et al., 2020). Perinatal BPA exposure also badly affected the maintenance of long-term memory on postnatal day 70, suggesting that neurodevelopmental toxicity due to perinatal BPA exposure might affect postnatal morphogenesis and functional development of the hippocampal dentate gyrus (Komada et al., 2020).

### Effect of BPA Exposure in Adult and Neonatal Animals *in vivo*

Adult BPA exposure has also been examined in earlier studies. In ovariectomized OVX adult rats, the application of only BPA (300 μg/kg body weight) via s.c. injection suppressed the spine-synapse density (spine rearrangement) to about 65% in the hippocampus though the injection of E2 (60 μg/kg) increased the spine-synapse density to about 160% (MacLusky et al., 2005). Co-application of BPA (300 μg/kg) with E2 (60 μg/kg) completely suppressed the E2-induced increase in the spine-synapse density. In the adult monkey, BPA exposure (50 μg/kg) using minipump over 4 weeks inhibited the E2-induced increase of spine synapse density in the hippocampus (Leranth et al., 2008).

Neonatal BPA exposure showed significant effects on other regions of the brain. In the rat cerebellum, the effect of neonatal BPA exposure on the development of Purkinje cells was investigated. Neonatal injection of BPA (500 μg/∼4 g/day for 4 days) into the cerebrospinal fluid of newborn rats resulted in the enhancement of dendritic outgrowth of Purkinje cells within 10 days (Shikimi et al., 2004). On this basis, BPA might have modulating effects on neuronal architecture also in the cerebellum.

### Bisphenol A Concentrations in Human and Animal Plasma

Possible risks of low-dose BPA exposure on humans are warned (Canadian Ministry of Health and Environment, 2008; Shelby, 2008). Low dosage of μg/kg BPA may induce nanomolar plasma concentration of BPA. The BPA concentrations in male human plasma and male rat plasma are approximately 1.5 ng/mL (6.5 nM) and 24.9 ng/mL (109 nM), respectively (Takeuchi and Tsutsumi, 2002; Takeuchi et al., 2004). From our earlier study, the BPA concentration in the rat hippocampus was approximately 14.7 ng/mL (64 nM) (Tanabe et al., 2012). The plasma BPA may reach the brain and be accumulated without significant detoxification.

## Conclusion

In conclusion, the current study demonstrates that the perinatal BPA exposure changed the hippocampal spine density of grownup rats with clear differences between males and females at different estrous cycle stages. Because hippocampal spines are involved in the mechanisms responsible for the formation of memory, these observations raise concerns regarding the potential impact of low-dose perinatal BPA exposure on impairment of healthy cognitive development and function.

## Data Availability Statement

The raw data supporting the conclusions of this article will be made available by the authors, without undue reservation.

## Ethics Statement

The animal study was reviewed and approved by the Laboratory Animal Committee of University of Tokyo.

## Author Contributions

SK conceived and designed the study. MO-I, MSo, HY, TK, SA, and YH conducted the experiments and analysis of the data. SK, MO-I, MSa, and YH wrote the manuscript. All authors provided feedback on the manuscript.

## Conflict of Interest

The authors declare that the research was conducted in the absence of any commercial or financial relationships that could be construed as a potential conflict of interest.

## Publisher’s Note

All claims expressed in this article are solely those of the authors and do not necessarily represent those of their affiliated organizations, or those of the publisher, the editors and the reviewers. Any product that may be evaluated in this article, or claim that may be made by its manufacturer, is not guaranteed or endorsed by the publisher.

## Abbreviations

E2: 17β-estradiol
ERα: estrogen receptor alpha
ERβ: estrogen receptor beta
ERRγ: estrogen receptor related protein gamma.
