# Supplementary figures and images for: Perinatal Exposure of Bisphenol A Differently Affects Dendritic Spines of Male and Female Grown-Up Adult Hippocampal Neurons
Source: Front Neurosci. 2021 Sep 20;15:712261. doi: 10.3389/fnins.2021.712261 (PMC8488347; doi:10.3389/fnins.2021.712261)

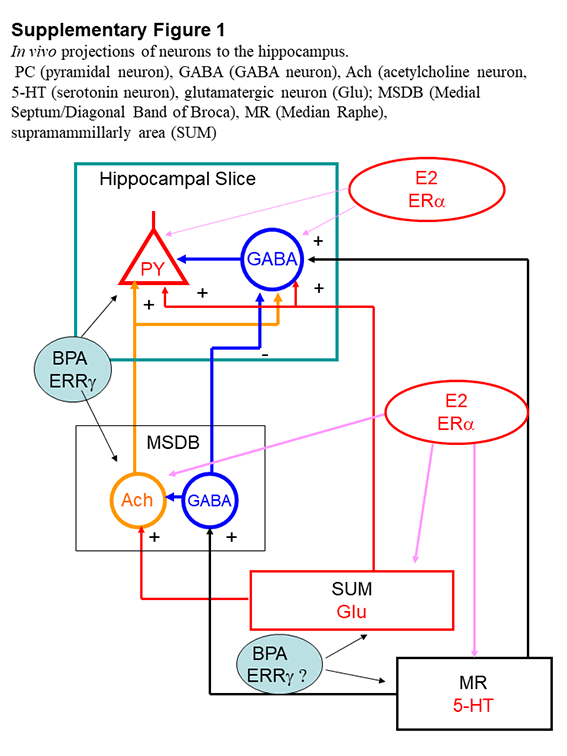

Supplement: Supplementary file 1 [file Image_1.tif]
